# Comparison of the Acute Effects of Auricular Vagus Nerve Stimulation and Deep Breathing Exercise on the Autonomic Nervous System Activity and Biomechanical Properties of the Muscle in Healthy People

**DOI:** 10.3390/jcm14041046

**Published:** 2025-02-07

**Authors:** Çağıl Ertürk, Ali Veysel Özden

**Affiliations:** 1 Faculty of Health Sciences, Department of Physiotherapy and Rehabilitation, Istanbul Gelisim University, Avcılar, Istanbul 34315, Türkiye; 2 Faculty of Health Sciences, Department of Physiotherapy and Rehabilitation, Bahcesehir University, Beşiktaş, Istanbul 34353, Türkiye; aliveysel.ozden@bau.edu.tr

**Keywords:** auricular vagus nerve stimulation, deep breathing exercises, heart rate variability, muscle relaxation, muscle tone

## Abstract

**Background/Objectives:** We aimed to examine the acute effects of deep breathing exercise and transcutaneous auricular vagus nerve stimulation (taVNS) on autonomic nervous system activation and the characteristics of certain muscle groups and to compare these two methods. **Methods:** 60 healthy adults between the ages of 18 and 45 were randomly divided into two groups to receive a single session of taVNS and deep breathing exercises. Acute measurements of pulse, blood pressure, perceived stress scale, autonomic activity, and muscle properties were performed before and after the application. **Results:** A significant decrease was detected in the findings regarding the perceived stress scale, pulse, and blood pressure values as a result of a single session application in both groups (*p* < 0.05). In addition, it was determined that the findings regarding autonomic measurement values increased in favor of the parasympathetic nervous system in both groups (*p* < 0.05). In measurements of the structural properties of the muscle, the stiffness values of the muscles examined in both groups decreased (*p* < 0.05), while the findings regarding relaxation increased (*p* < 0.05), except for the masseter in the deep breathing (DB) group. As a result of the comparative statistical evaluation between the groups, the increase in parasympathetic activity was found to be greater in the DB group according to root mean square of differences in successive RR intervals (RMSSD), the percent of differences in adjacent RR intervals > 50 ms (pNN50), and stress index parameters (*p* < 0.05). In the measurements made with the Myoton^®^PRO device, the increase in the relaxation value was higher in the gastrocnemius muscle of the VNS group (*p* < 0.05). **Conclusions:** It has been observed that both methods can increase parasympathetic activity and muscle relaxation in healthy people in a single session. However, DB appears to be slightly superior in increasing parasympathetic activity, and VNS appears to be slightly superior in increasing relaxation.

## 1. Introduction

The autonomic nervous system (ANS) is responsible for the regulation and integration of internal organ functions through the mutual actions of its sympathetic and parasympathetic branches. An evaluation of ANS functions is important to obtain information about the status of wellness or disease, and the activity of ANS can be affected by several methods such as deep breathing, valsalva maneuver, handgrip, and the cold pressor test [1]. Activity dysfunctions in the ANS have been associated with various clinical disorders such as heart failure, inflammatory bowel disease, and chronic pain syndromes, and it has been reported that this condition is mostly related to the lack of parasympathetic activity and relatively higher sympathetic activity [2]. Therefore, the regulation of ANS activity may contribute to recovery. Breathing exercises seem to increase parasympathetic activity assessed by heart rate variability (HRV) indexes even in one session. Simultaneous anxiety and stress levels may accompany this decline [3]. Deep breathing in diabetic patients can also decrease glycosylated hemoglobin levels and hypertension due to parasympathetic activity enhancement as shown by HRV [4]. Another new method to modulate the ANS activity is vagus nerve stimulation (VNS), which can be performed also non-invasively. VNS is a medical treatment method that can be used in the treatment of diseases such as epilepsy, depression, and migraine. VNS works by applying electrical impulses to the vagus nerve and can be applied to both ears [5]. Transcutaneous auricular vagus nerve stimulation (taVNS) provides an increase in parasympathetic activity and significantly reduces the incidence of the firing of sympathetic fibers [6]. There are few studies on the effects of deep breathing and VNS on muscle tone, and the results are inconclusive. Brief breathing exercises do not substantially affect muscle tension under psychological stress [7]. However, deep breathing exercises can reduce muscle tone by increasing parasympathetic activity. Similarly, taVNS appears to both reduce muscle tone and alter autonomic activity [8]. In this study, we hypothesized that taVNS and deep breathing exercises may increase parasympathetic activity and reduce muscle tone in healthy participants. So, we aimed to compare the effects of both methods and see their superiority over each other.

## 2. Materials and Methods

### 2.1. Study Design

This study was conducted at the Physical Therapy Laboratory of the Faculty of Health Sciences, Istanbul Gelisim University, between February and August 2022, in accordance with the rules of the Declaration of Helsinki. Before starting this study, ethics committee approval dated 6 January 2022 and numbered 2022/1 was obtained from Istanbul Yeni Yuzyil University Scientific Research and Publication Ethics Committee. The participants were asked to sign the informed consent form before participating in this study. The clinical trial registration number was NCT06740032.

### 2.2. Participants

Volunteer individuals participating in this study were randomly divided into two groups to receive “vagus nerve stimulation” and “deep breathing exercises”. Immediately before and after the single-session applications, measurements of ANS activity and muscle properties were recorded in both groups, and the obtained values were compared between groups. Publication approval was obtained from the individuals participating in this study. This study is a randomized prospective experimental study without a control group. Individuals were divided into two groups according to their order of arrival. The researcher informed the individual whose group was randomly determined which method would be applied.

### 2.3. Study Protocol

This study included 60 healthy participants, 30 in each group, aged between 18 and 45 years, who could read and write Turkish. The inclusion criteria were as follows: having no known acute or chronic disease, and no previous treatment with taVNS and deep breathing. All participants included in this study were selected from those who were at an intermediate level according to the International Physical Activity Fitness Survey (IPAQ). In this study, IPAQ short form and the one-on-one interview method were applied to determine the physical activity levels of people. The exclusion criteria were previous vagotomy, myocardial infarction or arrest, cardiac conduction disorders, intracranial hemorrhage, history of cancer and lung diseases, mental illness, using any medication supplement, and pregnancy during the study period. After the evaluation of 60 participants, a statistical analysis of the data was started. This study was started and completed as planned.

### 2.4. Assessment

#### 2.4.1. Vagus Nerve Stimulation

**Figure 1:**
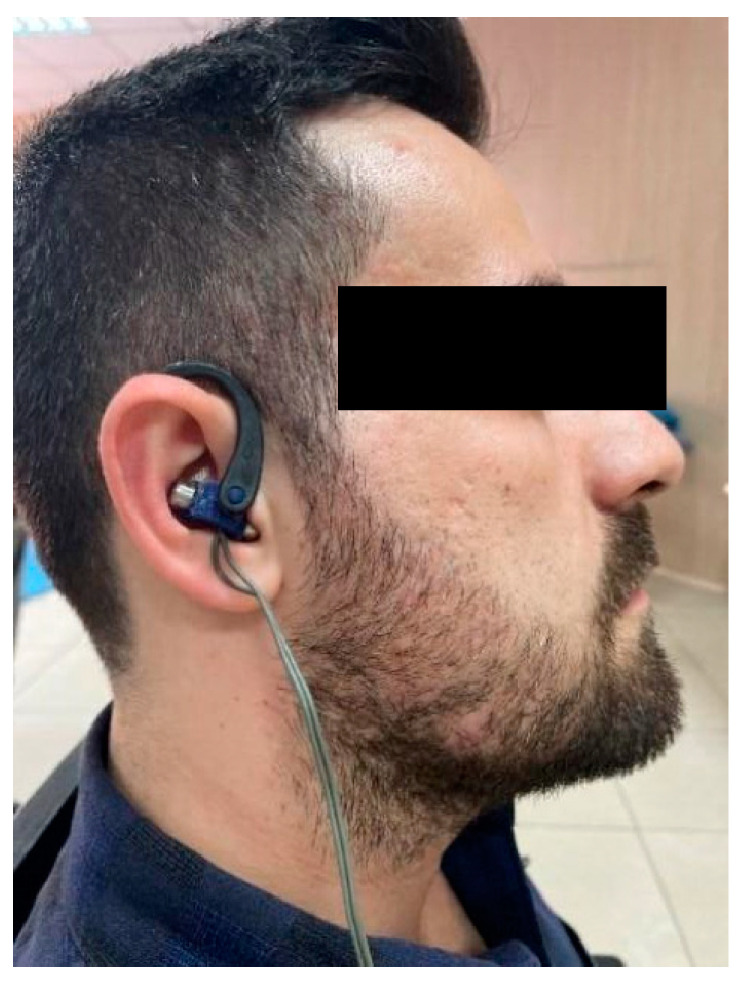
VNS right earset placement.

Participants in the VNS group were applied stimulation for 20 min with a frequency of 10 Hz and a pulse width of 300 µs. The earsets were placed in line with the concha and tragus. (Figure 1) For in-ear TaVNS, bilateral earsets (Copyright Vagustim, 2024, Vagustim Health Technologies LLC, Newark, DE, USA) with a surface area of 36 square millimeters were used to stimulate the tragus and concha. The Vagustim v1 device was the preferred stimulator (Copyright Vagustim, 2024, Vagustim Health Technologies LLC, Newark, DE, USA).

#### 2.4.2. Deep Breathing Exercise

The definition of slow, deep breathing is expressed in its broadest sense as slower than the typical adult rate of 12–15 breaths per minute. Various yogic techniques target a specific frequency of 6 breaths per minute (0.1 Hz). This frequency maximizes heart rate variability (HRV) [9]. Participants performed deep breathing exercises for 9 min in a semi-sitting position, with one hand on the diaphragm and the other on the thorax, in a position where they felt comfortable, followed by relaxation exercises for 2 min, and then breathing exercises again for 9 min. Slow deep breathing exercises were applied with an inspiration time of 3 s and an expiration time of 7 s, resulting in 6 breaths in 1 min.

### 2.5. Outcome Measures

#### 2.5.1. Primary Outcome Measures

In this study, primary outcome measures were HRV results and muscle properties such as stiffness and relaxation.

##### Analysis of the Autonomic Nervous System

Measurements were made using self-gel electrodes. One end of the e-motion faros device was connected to the electrode just below the participants’ right clavicle, the other end was connected to the bottom of the left clavicle, and the third end was connected to the electrode just above the lowest left rib. Measurements were made in a semi-sitting position where the individual felt comfortable. Participants were told not to drink coffee, tea, or smoke cigarettes for at least 2 h before the measurements, not to drink alcohol for at least 24 h, and not to exercise in a way that would increase their heart rate. Measurements were taken 2–4 h after (neither hungry nor full) the last meal. Those who had insomnia the night before were measured on another day. After 30 min of rest, autonomic measurements were taken before and after taVNS and deep breathing exercise methods. Pulse and blood pressure measurements were made after HRV in order not to affect it. During the 5 min HRV measurement, the participant was asked not to move or talk and to sit comfortably with their eyes closed. After taVNS and breathing exercise application, the measurement made with the e-motion faros device was repeated in the same way, and the pulse and blood pressure were measured again. The measurement records made with the e-motion faros device were later analyzed using kubios software (3.5.0 Version), and the following values were obtained: sympathetic nervous system (SNS) index, parasympathetic nervous system (PNS) index, stress index, low frequency/high frequency ratio (LF/HF), PNN50, and RMSSD.

- High frequency (HF): It shows more parasympathetic activity.
- leaseLow frequency (LF): It indicates more sympathetic activity.
- RMSSD: It is the root mean square of consecutive R-wave peak to R-wave peak interval (RRI) differences. It reflects beat-to-beat variance in heart rate (HR), indicating parasympathetic activity.
- Stress index: It indicates sympathetic activity.
- SNS index: It indicates sympathetic activity.
- PNS index: It indicates parasympathetic activity.
- PNN50: It refers to the division of the number of normal to normal R-R (NN) intervals differing by >50 ms from the preceding interval (NN50) by the total number of NN intervals. It indicates parasympathetic activity [10].

##### Analysis of the Structural Features of the Muscles

**Figure 2:**
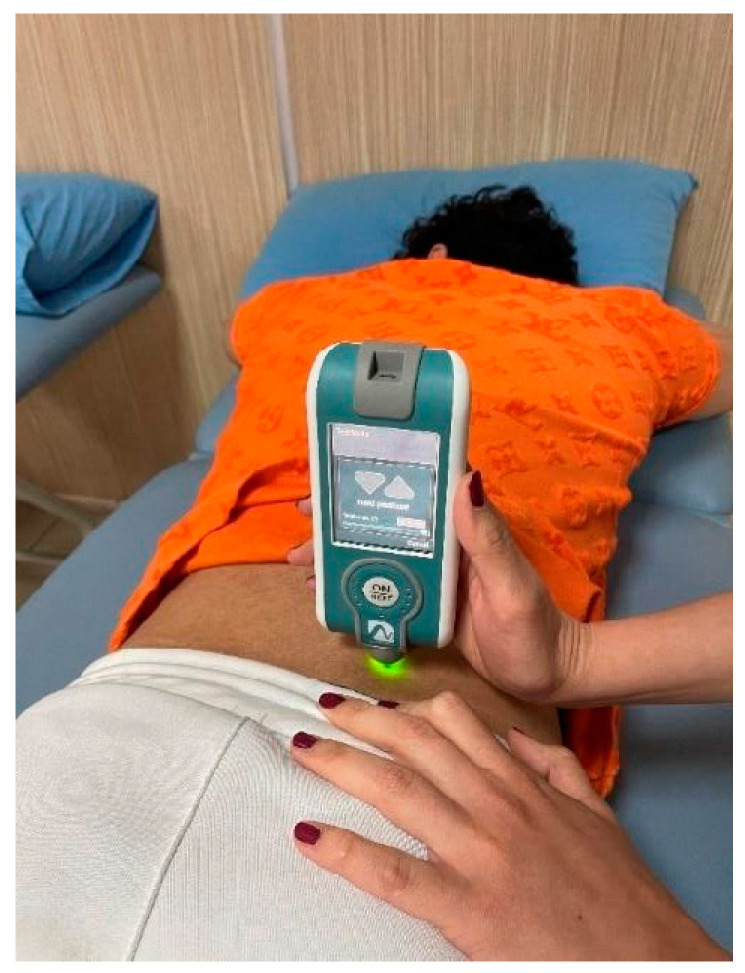
Application of Myoton^®^PRO to m. erector spinae.

The Myoton^®^PRO device (Myoton AS, Tallinn, Estonia) is a reliable and portable digital palpation device that can provide a better understanding of the relationship of biomechanical properties such as tone, stiffness, and flexibility to muscle health and physical condition. The application of Myoton^®^PRO: The masseter, trapezius (upper part), erector spinae, gastrocnemius, and biceps brachii muscles of the individuals included in this study were measured bilaterally. The individuals were allowed to lie relaxed in the supine position for 10 min to allow their muscles to rest, and the trapezius (upper part) in the sitting position, erector spinae and gastrocnemius muscles in the prone position, biceps brachii and masseter muscles in the supine position were evaluated bilaterally (Figure 2).

The specific evaluation position and point of the muscle was made according to the criteria and positions recommended by the Myoton^®^PRO team (Myoton^®^PRO, https://www.myoton.com/applications/, Date of access: 1 May 2022). The device was placed on each muscle surface in an upright position, and the tip of the probe was placed on the motor point of the measured muscle. Dynamic stiffness (S) and relaxation (R) data were used for muscle characteristics. The average bilateral measurements of each muscle group were evaluated.

#### 2.5.2. Secondary Outcome Measures

Participants’ pulse and blood pressure values and perceived stress scale results before and after the application were recorded. Pulse and blood pressure were measured with the Visomat brand device while the participants sat quietly and motionless in a comfortable position for 60 s. In order to obtain reliable data, participants subjected to the application were informed that they should not consume tea, coffee, or cigarettes at least 2 h before the measurements started and until the end of the procedure, that they also should not have consumed alcohol in the last 24 h, and that it was important to come to the measurement in comfortable clothes.

### 2.6. Statistical Analysis

Sample size was calculated by using G-Power. When T test family and Mann–Whitney U Test were used: repeated measures, within–between interaction test, effect size: 0.80, beta level: 0.20, power (1-β): 0.80, number of groups: 2, number of measurements: 2, correction among repeated measures: 0.5 was selected, and the total sample size was calculated as 60 participants (30 participants in each group). All the statistical analyses were performed using IBM SPSS 22.0 and 25.0 softwares. The descriptive statistics of the variables used are n and frequency in categorical data; in continuous data, mean, standard deviation, and minimum and maximum values were used. First of all, it was examined whether continuous variables had normal distribution according to the Shapiro–Wilk test. Nonparametric tests were applied for variables that did not have a normal distribution (*p* < 0.05). The Wilcoxon Signed-Rank Test was used to measure the difference between the means of two dependent groups, and the Mann–Whitney U test was used to measure the difference between the means of two independent groups. In all analyses, the significance level was given as 0.05. The nonparametric ANCOVA method was applied to compare the “DB” and “taVNS” groups in terms of the difference between the second and first measurements. In the model used, the difference between the two measurements (second measurement—first measurement) was set as the dependent variable, while the first measurement result was included as a covariate. For this purpose, the procedure outlined in the manual of the program for a nonparametric or rank analysis of covariance (Quade’s test) was followed using IBM SPSS 25.0 software.

## 3. Results

The gender distribution of the participants in the VNS and DB groups participating in this study is shown in the table below. It was determined that participants in the VNS group were predominantly female (*p* < 0.05) (Table 1).

Considering the demographic information of the individuals, there was no significant difference in the average height and weight between the two groups, while the average age in the VNS group was found to be higher (*p* < 0.05) (Table 2).

It was determined that there were significant decreases in the perceived stress scale, pulse, systolic and diastolic blood pressure values in both the DB and VNS groups compared to the first measurement average (*p* < 0.05) (Table 3).

Considering the HRV values, it was revealed that there was a statistically significant increase in the PNS index and RMSSD and PNN50 values in the DB group, while there was a significant decrease in the SNS index and stress index values (*p* < 0.05). Although there was a decrease in the LF/HF ratio, no statistical significance was observed (*p* > 0.05). In the VNS group, while there was a statistically significant increase in the PNS index and RMSSD and PNN50 values, it was revealed that there was a significant decrease in the perceived stress scale, SNS index, and stress index values (*p* < 0.05). Although there was a decrease in the LF/HF value, no statistical significance was observed (*p* > 0.05) (Table 3).

In the DB group, in the measurements of the structural properties of the muscle, a decrease was observed in the measurement values of all muscles in the stiffness parameter (*p* < 0.05), while a statistically significant increase was observed in the relaxation parameter in all muscles except the masseter muscle, while the increase in the masseter was not found to be statistically significant (*p* > 0.05). In the VNS group, while a decrease was observed in the measurement values of all muscles in the stiffness parameter (*p* < 0.05), a statistically significant increase was observed in the relaxation parameter in all muscles (*p* < 0.05) (Table 3).

**Figure 3:**
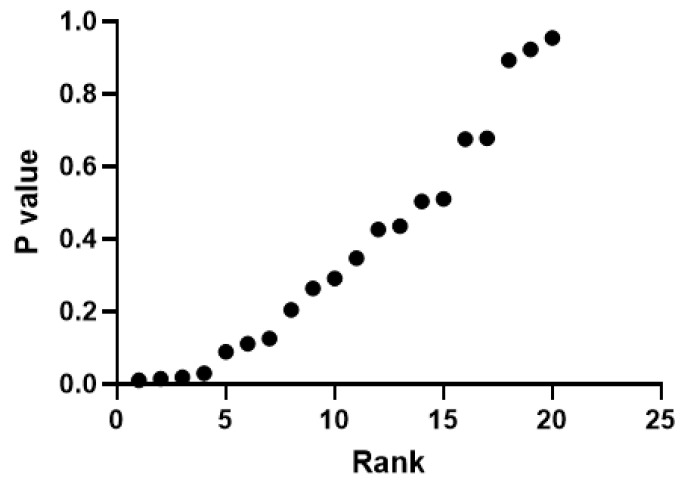
Evaluation of the false discovery rate of the results obtained from intergroup analysis.

There was no significant difference between groups in terms of perceived stress scale, pulse and systolic/diastolic blood pressure, LF/HF ratio, and PNS and SNS index values (*p* > 0.05). When the RMSSD, PNN50, and stress index results were compared, it was found that the DB group was statistically higher than the VNS group (*p* < 0.05) (Table 4) (Figure 3).

In the intergroup comparison, a statistically significant increase was detected in the relaxation value of the gastrocnemius muscle in the VNS group (*p* < 0.05) (Table 4).

## 4. Discussion

In this study, in which we compared the effects of taVNS and deep breathing exercises on healthy people, a significant decrease was detected in the findings regarding the perceived stress scale, pulse, and blood pressure values as a result of a single session of application in both groups, in accordance with the literature data [4,11]. In addition, the findings regarding HRV values in both groups developed in favor of the parasympathetic nervous system, again in line with the literature [3,6]. When the effects on the structural properties of various muscle groups were examined in this study, a decrease in the stiffness and an increase in the relaxation values of the muscles was observed with both applications. Clancy et al. (2014) stated that single-session stimulation of the tragus part of the ear using 200 µs pulses at 30 Hz, 10–50 mA provided an increase in parasympathetic activity and reduced the incidence of the firing of sympathetic fibers [6]. In this study, 10 Hz, 300 microsecond pulse width stimulation was applied bilaterally to the tragus and concha, and it was also revealed that parasympathetic activity increased.

ANS modulation via taVNS is proposed as a non-invasive therapeutic strategy for the treatment of cardiovascular diseases [12]. Billman (2013) gave a different perspective to the idea that the LF/HF ratio measures “sympatho-vagal balance”. He reported that LF does not only reflect SNS activity, and half of the variability in this frequency band originates from the PNS [13]. It is also stated by other researchers that there is uncertainty about the contributions of PNS and SNS to the LF/HF ratio [10]. In this study, systolic/diastolic blood pressure and pulse measurement values decreased significantly after a single session of taVNS. Moreover, there was no significant change in the LF/HF ratio as a result of a single session of taVNS. While other HRV parameters changed in favor of parasympathetic activity, the lack of change in the LF/HF ratio may have been related to the measurement time (5 min of measurement), the number of participants (60 people), and their characteristics (healthy people). As a matter of fact, Soltani et al. (2023) evaluated the effect of taVNS on the LF/HF ratio in fifteen studies conducted with 380 healthy participants, and in seven studies, it was reported that there was no significant difference in the LF/HF ratio between the VNS group and the control group. In the same study, it was stated that there was a high correlation between the level of autonomic dysfunction and the response to taVNS [14]. In this study, conducted on healthy individuals, no significant change was observed in the LF/HF ratio, which is consistent with previous studies.

The study of Ferstl et al. (2022), in which 90 min of taVNS was performed from one ear (right or left) by randomization in 82 healthy participants, reported that taVNS improved positive mental state but was only effective in the acute phase after stimulation [15]. Cook et al. (2021), examined virtual reality-based deep breathing exercises in head injury rehabilitation. Fifteen participants were given 5 min-deep breathing exercises with virtual reality applications, and participants reported a decrease in stress, tension, fatigue, and confusion following the deep breathing exercise [16]. In this study, we found that short-term deep breathing practices led to a decrease in perceived stress scales. Deep slow breathing increases vagus nerve activity, which is indexed by HRV [17]. Jensen et al. (2022) compared the effect of taVNS and DB on vagal tone in healthy participants and patients with rheumatoid arthritis (RA) or systemic lupus erythematosus (SLE), and vagal tone was evaluated using HRV parameters. In total, 30 min of DB and 30 min of taVNS were applied to 42 healthy participants and 52 patients on separate days. While DB was associated with the highest increase in HRV parameters in healthy participants, both applications caused an increase in HRV parameters in favor of parasympathetic activity in patients [18]. As in this study, DB exercises seem to be more effective than taVNS in increasing parasympathetic activity in healthy individuals. However, this situation seems to change in patients with autonomic dysfunction.

Studies have shown that slow breathing can reduce the stress response by encouraging the body to relax [19]. Kampusch et al., in their study, applied taVNS to a patient with cervical dystonia who was resistant to treatment for 20 months. As a result of the treatment, it was determined that there was a decrease in muscle tone in the right and left trapezius muscles [8]. However, there are also studies showing that a single session of taVNS increases muscle activation in healthy people [20]. We think that measuring muscle activation during muscle contraction with electromyography in their study of Konakoglu et al. may cause such a difference to emerge. In this study, it was determined that as a result of a single session of taVNS, there was a statistically significant decrease in the stiffness values of muscles measured with the Myoton^®^PRO device and a significant increase in the relaxation parameter. The results obtained are consistent with those of Kampusch et al. (2015), who stated that taVNS reduces muscle tone by increasing parasympathetic activity [8]. In their study by Moon et al. (2018) using Myoton^®^PRO, they stated that the sitting position with lumbar lordosis assist caused a decrease in upper trapezius muscle tone in participants with head anterior tilt [21]. In another similar study using Myoton^®^PRO, Kim (2021) determined that there was a correlation between the low mixing ability index of the masseter muscle and high muscle tone and low flexibility [22]. Skeletal muscles normally receive no parasympathetic innervation, and the autonomic effects appear to be exclusively sympathetic in origin, mediated either by the neurally released noradrenaline or indirectly through circulating adrenaline released in the blood by the adrenal medulla. Thus, sympathetic activity can increase muscle contractility and excitability [23]. So, it can be said that elevated parasympathetic and decreased sympathetic activity can reduce tension in the muscles and lead to relaxation as seen in this study for both applications. We found that taVNS was superior only in relaxation of the gastrocnemius muscle. This difference may be considered minor, given similar results for other muscles.

This study has some limitations. The most important limitation is that the applications to individuals in both groups were carried out in a single session. In addition, the average age and gender distribution of the participants in the VNS and DB groups are not homogeneous (Table 1 and Table 2). Relevant measurements were performed immediately after the application in the VNS and DB groups. No side effects were observed due to the applications in this study. Randomized prospective studies with longer duration, using additional measurement methods, and conducted in healthy individuals or different patient populations are needed to develop data regarding autonomic responses and how long the changing properties of the muscle last.

## 5. Conclusions

In conclusion, we found that a single-session application of taVNS and DB was effective in changing HRV and the biomechanical properties of the muscle in healthy individuals. However, deep breathing exercises were found to be slightly more effective in increasing parasympathetic activity than taVNS. Deep breathing probably has a direct effect on the ANS (affecting both efferent and afferent fibers), but taVNS stimulates only afferent fibers, which may have made deep breathing superior in terms of ANS activity modification. However, taVNS increased relaxation more for a single muscle, but we think this can be neglected.

## Figures and Tables

**Table 1:** Comparison of intergroup differences in gender descriptive statistics evaluation.

Gender	VNS		DB		Total		Pearson Chi-Square	*p*
	n	% İntragroup	n	% İntragroup	n	% İntergroup		
Male	5	16.67	15	50.00	20	33.33	7.500	0.006
Female	25	83.33	15	50.00	40	66.67		
Total	30	100	30	100	60	100		

**Table 2:** Comparison of intergroup differences in age, height, and weight parameters.

N = 60	DB N = 30			VNS N = 30		
	Mean ± SD	Z/t	*p*	Mean ± SD	Z/t	*p*
Height (cm)	171.97 ± 9.34	−0.074	0.941	171.70 ± 8.17	−0.074	0.941
Weight (kg)	66.60 ± 12.54	−0.429	0.668	68.40 ± 13.22	−0.429	0.668
Age	24.70 ± 4.50	−2.859	**0.004**	31.27 ± 8.23	−2.859	**0.004**

Bold means *p* < 0.05.

**Table 3:** Comparison of intragroup differences in blood pressure, pulse, and measurement of autonomic and structural properties of muscle parameters.

		DB			VNS		
		Mean ± SD	Z/t	*p*	Mean ± SD	Z/t	*p*
Perceived stress scale	1st measurement	27.20 ± 7.29	3.892	0.001 **	26.10 ± 8.31	2.443	0.021 **
	2nd measurement	25.97 ± 7.26			24.80 ± 7.81		
Pulse	1st measurement	78.53 ± 9.73	4.556	0.000 **	79.53 ± 9.90	6.009	0.000 **
	2nd measurement	74.00 ± 9.74			73.50 ± 8.11		
Systolic pressure	1st measurement	118.23 ± 11.31	5.245	0.000 **	119.73 ± 13.74	6.394	0.000 **
	2nd measurement	112.33 ± 12.47			112.37 ± 13.76		
Diastolic pressure	1st measurement	73.40 ± 10.03	3.267	0.003 **	76.00 ± 12.03	4.906	0.000 **
	2nd measurement	70.13 ± 10.08			70.50 ± 13.57		
SNS index	1st measurement	0.24 ± 1.01	6.041	0.000 **	0.74 ± 0.95	7.75	0.000 **
	2nd measurement	−0.48 ± 1.16			0.02 ± 0.79		
PNS index	1st measurement	0.35 ± 1.32	−4.33	0.000 *	−0.46 ± 1.13	−4.76	0.000 *
	2nd measurement	9.34 ± 17.59			0.55 ± 1.74		
RMSSD	1st measurement	53.94 ± 30.71	−4.78	0.000 *	47.03 ± 34.88	−4.73	0.000 *
	2nd measurement	232.60 ± 421.98			75.83 ± 59.47		
Stress index	1st measurement	8.25 ± 3.07	8.519	0.000 **	9.39 ± 3.65	−4.58	0.000 *
	2nd measurement	4.79 ± 2.22			6.61 ± 2.65		
PNN50	1st measurement	21.70 ± 13.31	−6.23	0.000 **	13.23 ± 15.54	−3.96	0.000 *
	2nd measurement	40.01 ± 21.46			20.63 ± 16.84		
LF/HF	1st measurement	1.37 ± 1.62	−1.29	0.199	2.56 ± 2.44	−0.83	0.405
	2nd measurement	1.14 ± 0.62			2.60 ± 2.39		
Stiffness-trapezius muscle	1st measurement	267.18 ± 33.88	5.784	0.000 **	259.43 ± 44.90	−4.38	0.000 *
	2nd measurement	249.70 ± 31.19			244.45 ± 43.59		
Stiffness-erector spinae muscle	1st measurement	235.53 ± 87.88	−4.54	0.000*	192.15 ± 46.10	−3.67	0.000 *
	2nd measurement	235.53 ± 87.88			185.92 ± 43.91		
Stiffness- gastrocnemius muscle	1st measurement	262.80 ± 53.84	3.466	0.000 **	236.68 ± 38.18	5.163	0.000 **
	2nd measurement	249.10 ± 50.39			227.13 ± 39.87		
Stiffness-biceps brachii muscle	1st measurement	243.65 ± 36.29	−4.78	0.000 *	229.68 ± 31.29	−4.63	0.000 *
	2nd measurement	230.60 ± 37.59			216.18 ± 23.66		
Stiffness-masseter muscle	1st measurement	328.25 ± 88.46	3.444	0.000 **	362.68 ± 61.49	3.533	0.001 **
	2ndmeasurement	313.47 ± 76.18			343.65 ± 57.96		
Relaxation-trapezius muscle	1st measurement	18.37 ± 1.94	−3.97	0.000 *	18.66 ± 1.99	−4.47	0.000 **
	2nd measurement	19.09 ± 1.98			19.69 ± 2.02		
Relaxation-erector spinae muscle	1st measurement	22.98 ± 5.22	−4.57	0.000 *	26.53 ± 3.98	−4.25	0.000 *
	2nd measurement	24.23 ± 4.42			27.05 ± 3.89		
Relaxation-gastrocnemius muscle	1st measurement	19.04 ± 4.05	−2.24	0.014 *	22.46 ± 3.56	−5.71	0.000 **
	2nd measurement	19.57 ± 3.93			23.63 ± 3.59		
Relaxation-biceps brachii muscle	1st measurement	19.60 ± 1.75	−4.92	0.000 *	20.84 ± 2.26	−4.1	0.000 *
	2nd measurement	20.60 ± 1.36			21.72 ± 1.79		
Relaxation-masseter muscle	1st measurement	15.84 ± 2.90	−1.53	0.138	15.67 ± 2.73	−4.98	0.000 **
	2nd measurement	16.18 ± 2.70			16.39 ± 2.70		

Note: * Wilcoxon Signed-Rank Test, ** Paired Sample *t*-Test. Abbreviations: DB, deep breathing; VNS, vagus nerve stimulation. Bold means *p* < 0.05.

**Table 4:** Comparison of intergroup differences in blood pressure, pulse, and measurement of autonomic and structural properties of muscle parameters.

	DB	VNS	t	*p*
	Mean ± SD	Mean ± SD		
Perceived stress scale	−1.23 ± 1.74	−1.30 ± 2.91	−0.663	0.510
Pulse	−4.53 ± 5.45	−6.03 ± 5.49	−1.729	0.089
Diastolic pressure	−3.27 ± 5.48	−5.50 ± 6.14	−1.281	0.205
Systolic pressure	−5.90 ± 6.16	−7.37 ± 6.31	−0.097	0.923
RMSSD	178.67 ± 413.87	28.79 ± 36.28	−2.221	**0.030**
Stress index	−3.45 ± 2.22	−2.77 ± 2.63	−2.417	**0.019**
PNN50	18.31 ± 16.10	7.40 ± 9.14	−2.518	**0.015**
LF/HF	−0.23 ± 1.60	0.04 ± 2.29	−1.555	0.125
SNS index	−0.73 ± 0.66	−0.71 ± 0.50	−1.136	0.893
PNS index	8.99 ± 17.25	1.008 ± 1.08	−1.129	0.264
Stiffness-trapezius muscle	−17.48 ± 16.55	−14.98 ± 17.23	−0.672	0.504
Stiffness-erector spinae muscle	−26.72 ± 48.77	−6.23 ± 9.05	−0.785	0.435
Stiffness-gastrocnemius muscle	−13.70 ± 21.65	−9.55 ± 10.13	−1.065	0.291
Stiffness-biceps brachii muscle	−13.05 ± 9.12	−13.50 ± 19.42	−0.422	0.675
Stiffness-masseter muscle	−14.78 ± 23.51	−19.03 ± 29.51	−0.801	0.426
Relaxation-trapezius muscle	0.73 ± 1.00	1.03 ± 1.26	−0.949	0.347
Relaxation-erector spinae muscle	1.25 ± 1.49	0.52 ± 0.58	−1.616	0.111
Relaxation-gastrocnemius muscle	0.53 ± 1.30	1.17 ± 1.13	−2.346	**0.010**
Relaxation-biceps brachii muscle	0.99 ± 1.11	0.88 ± 1.11	−0.057	0.955
Relaxation-masseter muscle	0.34 ± 1.23	0.72 ± 0.79	−0.418	0.678

Note: DB, deep breathing; VNS, vagus nerve stimulation. Bold means *p* < 0.05.

## Data Availability

Data are available on request from the authors.

## References

[1] Jha R.K., Acharya A., Nepal O. (2018). Autonomic Influence on Heart Rate for Deep Breathing and Valsalva Maneuver in Healthy Subjects. JNMA J. Nepal. Med. Assoc..

[2] Farmer A.D., Albu-Soda A., Aziz Q. (2016). Vagus nerve stimulation in clinical practice. Br. J. Hosp. Med..

[3] Magnon V., Dutheil F., Vallet G.T. (2021). Benefits from one session of deep and slow breathing on vagal tone and anxiety in young and older adults. Sci. Rep..

[4] Sridhar B., Haleagrahara N., Bhat R., Kulur A.B., Avabratha S., Adhikary P. (2010). Increase in the heart rate variability with deep breathing in diabetic patients after 12-month exercise training. Tohoku J. Exp. Med..

[5] Butt M.F., Albusoda A., Farmer A.D., Aziz Q. (2020). The anatomical basis for transcutaneous auricular vagus nerve stimulation. J. Anat..

[6] Clancy J.A., Mary D.A., Witte K.K., Greenwood J.P., Deuchars S.A., Deuchars J. (2014). Non-invasive vagus nerve stimulation in healthy humans reduces sympathetic nerve activity. Brain Stimul..

[7] Liang W.-M., Xiao J., Ren F.-F., Chen Z.-S., Li C.-R., Bai Z.-M., Rukšenas O. (2023). Acute effect of breathing exercises on muscle tension and executive function under psychological stress. Front. Physiol..

[8] Kampusch S., Kaniusas E., Széles J.C. (2015). Modulation of Muscle Tone and Sympathovagal Balance in Cervical Dystonia Using Percutaneous Stimulation of the Auricular Vagus Nerve. Artif. Organs.

[9] Peng C.K., Henry I.C., Mietus J.E., Hausdorff J.M., Khalsa G., Benson H., Goldberger A.L. (2004). Heartrate Dynamics during three forms of meditation. Int. J. Cardiol..

[10] Shaffer F., Ginsberg J.P. (2017). An overview of heart rate variability metrics and norms. Front. Public Health.

[11] Kutlu N.R. (2019). The Effect of Auricular Vagus Nerve Stimulation on Pain and Quality of Life in Patients with Fibromyalgia Syndrome. Master’s Thesis.

[12] He B., Lu Z., He W., Huang B., Jiang H. (2016). Autonomic modulation by electrical stimulation of the parasympathetic nervous system: An emerging intervention for cardiovascular diseases. Cardiovasc. Ther..

[13] Billman G.E. (2013). The LF/HF ratio does not accurately measure cardiac sympatho-vagal balance. Front. Physiol..

[14] Soltani D., Azizi B., Sima S., Tavakoli K., Mohammadi N.S.H., Vahabie A.-H., Akbarzadeh-Sherbaf K., Vasheghani-Farahani A. (2023). A systematic review of the effects of transcutaneous auricular vagus nerve stimulation on baroreflex sensitivity and heart rate variability in healthy subjects. Clin. Auton. Res..

[15] Ferstl M., Teckentrup V., Lin W.M., Kräutlein F., Kühnel A., Klaus J., Walter M., Kroemer N.B. (2022). Non-invasive vagus nerve stimulation boosts mood recovery after effort exertion. Psychol. Med..

[16] Cook N.E., Huebschmann N.A., Iverson G.L. (2021). Safety and tolerability of an innovative virtual reality-based deep breathing exercise in concussion rehabilitation: A pilot study. Dev. Neurorehabilit..

[17] De Couck M., Caers R., Musch L., Fliegauf J., Giangreco A., Gidron Y. (2019). How breathing can help you make better decisions: Two studies on the effects of breathing patterns on heart rate variability and decision-making in business cases. Int. J. Psychophysiol..

[18] Jensen M.K., Andersen S.S., Andersen S.S., Liboriussen C.H., Kristensen S., Jochumsen M. (2022). Modulating Heart Rate Variability through Deep Breathing Exercises and Transcutaneous Auricular Vagus Nerve Stimulation: A Study in Healthy Participants and in Patients with Rheumatoid Arthritis or Systemic Lupus Erythematosus. Sensors.

[19] Jerath R., Edry J.W., Barnes V.A., Jerath V. (2006). Physiology of long pranayamic breathing: Neural respiratory elements may provide a mechanism that explains how slow deep breathing shifts the autonomic nervous system. Med. Hypotheses.

[20] Konakoğlu G., Özden A.V., Solmaz H., Bildik C. (2023). The effect of auricular vagus nerve stimulation on electroencephalography and electromyography measurements in healthy persons. Front. Physiol..

[21] Moon J.-H., Jung J.-H., Hahm S.-C., Oh H.-K., Jung K.-S., Cho H.-Y. (2018). Effects of lumbar lordosis assistive support on craniovertebral angle and mechanical properties of the upper trapezius muscle in subjects with forward head posture. J. Phys. Ther. Sci..

[22] Kim H.E. (2021). Influential factors of masticatory performance in older adults: A cross-sectional study. Int. J. Environ. Res. Public Health.

[23] Roatta S., Passatore M. (2009). Autonomic effects on skeletal muscle. Encyclopedia of Neuroscience.

